# The pervasive nature of uncertainty—a qualitative study of patients with advanced cancer and their informal caregivers

**DOI:** 10.1007/s11764-017-0628-x

**Published:** 2017-07-18

**Authors:** Valerie Shilling, Rachel Starkings, Valerie Jenkins, Lesley Fallowfield

**Affiliations:** Sussex Health Outcomes Research and Education in Cancer, Brighton and Sussex Medical School, University of Sussex, Falmer, Brighton, BN1 9QG UK

**Keywords:** Qualitative, Cancer, Patients, Caregivers, Uncertainty

## Abstract

**Purpose:**

The aim of this study was to explore the impact of extended cancer survival on broader aspects of life and wellbeing such as occupational, financial and family life for patients with advanced cancer and their nominated informal caregivers.

**Methods:**

In-depth qualitative interviews were transcribed verbatim. A thematic framework was developed from an initial process of open coding and tested iteratively as new data were collected.

**Results:**

Twenty-four patient-caregiver dyads with advanced ovarian (9), melanoma (9) or lung cancer (6). Patients were aged 39–84 (median 62 years) and caregivers 19–85 (median 54 years). Caregivers were the partners/spouses (15), children (5), siblings (2) and friends (2) of patients. One particular theme, ‘uncertainty’, encompassed many issues such as planning for the future, providing for one’s family, employment and finances. Uncertainties were related to the timescale and trajectory of the disease and lack of control or ability to make plans. There were marked age effects. Accounts from within the same dyad often differed and patients and caregivers rarely discussed concerns with each other.

**Conclusions:**

Both patients and their informal caregivers were challenged by the uncertainties around living with advanced cancer and the lack of a defined trajectory. This impacted many diverse areas of life. Although distressing, dyads seldom discussed these concerns with each other.

**Implications for Cancer Survivors:**

Uncertainty is a recurrent issue for cancer survivors and their families impacting broad aspects of their lives and their ability to move forward; however, patients and caregivers in this study rarely discussed these concerns together. Uncertainty should be discussed periodically, together, and healthcare professionals could facilitate these discussions. The use of one or more ‘trigger questions’ in clinic appointments may provide an opportunity to start these dialogues.

**Electronic supplementary material:**

The online version of this article (doi:10.1007/s11764-017-0628-x) contains supplementary material, which is available to authorized users.

## Background

Progress into the biology underpinning many cancers and precision medicine has led to a dramatic increase in patients living with advanced cancer as a chronic condition. The management of these patients can be complex with some difficult decision-making around offering repeated further lines of treatment after progression that might control cancer but affect the quality of patients’ lives [1]. As increasing numbers of patients are living with, and living longer with advanced cancer, there is growing recognition of the importance of the quality of this survival: how the broader aspects of their lives are managed and impacted across the disease trajectory. When patients are living with advanced cancer, with no curative treatment, how do they and their families adjust to the uncertainty that surrounds the length and quality of their survival? Although some studies have highlighted how cancer affects employment, [2, 3], finances [4–6] and social roles and responsibilities such as parenting or being a caregiver to others [7–9], our understanding of the psychosocial sequelae of surviving longer with advanced cancer lags behind the scientific progress that has made it possible [1].

Patients rarely live in a social vacuum and cancer necessarily impacts on the whole family [10–12]. Informal caregivers frequently provide a significant amount of care and support for patients [13, 14] often alongside the other social and occupational roles and additional caregiving responsibilities that they may have [15–18].

The potential negative effects on patients and caregivers are likely to change over time as patients move through different phases of cancer treatment. Some researchers have conceptualised the cancer trajectory as having different critical moments reflecting patient and caregiver experiences of disease and treatment, rather than the biology of the disease itself, and note that these phases such as starting a new treatment often trigger changes in other areas of life and in the caregiver role [19, 20].

We designed the PROACT study (Patient Reported Outcomes impact of Age and Carer role demands associated with Treatment) recognising that patients and their families are continually adjusting to a fluid situation whilst trying to maintain their ‘real-world’ roles and responsibilities beyond cancer, such as caregiving responsibilities for a spouse or children/grandchildren, jobs and financial responsibilities. PROACT is a multi-phase project with the primary aim of developing and evaluating one self-report measure for patients and one for informal caregivers that would comprehensively assess these impacts on roles and responsibilities. These measures are intended to benefit future patients and caregivers by capturing these wider impacts of cancer and its treatment during the evaluation of new treatments in clinical trials. Additionally, these measures could be used clinically to aid treatment and intervention discussions.

In this paper, we present some of the qualitative findings from the first stage of measure development in the PROACT study; in-depth, semi-structured interviews with patients and their informal caregivers to inform item development. In the course of the analysis to fulfil the primary objective (i.e. the development of scale items), we developed a framework of 20 major themes and 33 subthemes. We also observed however ‘uncertainty’ repeatedly underpinning much of the patients’ and caregivers’ dialogue encompassing key areas of functional and emotional wellbeing. The most pertinent aspects varied, yet there was an underlying thread through all interviews that uncertainty was always present and destructive. It is this specific aspect of the analysis, the topic of uncertainty and the areas of its influence, which we unpack in this paper.

This is not a new topic in the cancer landscape. Qualitative studies have previously reported that uncertainty about the future can dominate the thoughts of patients and caregivers, but participants’ accounts have focused on the patient’s health, fears of recurrence and prognosis rather than broader life uncertainties [21–24]. Some studies however have highlighted that the difficulty of ‘not knowing’ went beyond health and prognosis, extending to wide-ranging areas of life such as making choices about jobs and careers or moving households [25, 26].

It is clear then that while uncertainty is not a new topic in cancer, it is one which continues to resonate with patients and their families [27]. Arguably, as novel treatments have resulted in a rapidly changing landscape for patients and caregivers living with cancer as a chronic condition, uncertainty may be even more pervasive and warrants revisiting. Several aspects of our study design help broaden our understanding of this important topic: the topic was explored with patient and caregiver dyads, allowing for consideration of different perspectives on the same situation. Patients nominated the person who was their main source of informal support reflecting impact more diversely than studies focused solely on partners as caregiver. In-depth qualitative interviews allowed patients and caregivers to intuitively discuss the pervasive nature of uncertainty using their own language and without specific prompt. This is in contrast to studies that have specifically asked about or measured levels of uncertainty as a means of linking to depression or anxiety for example and the tumour types and disease stage included in this study.

Kruizinga and colleagues [28, 29] describe the experience of having cancer as a contingent life event where the expected course of events is disrupted forcing patients and, by extension caregivers, to reinterpret and re-evaluate their lives and ultimately their underlying life goals. This provided us with a useful lens through which to view our PROACT interviews.

### Aims and objectives

In this paper, we describe one of the overarching themes from our interviews, that of ‘uncertainty’, and unpick how this construct pervaded many different aspects of patients’ and caregivers’ lives. We explore how the general concept varied within the accounts of patient-caregiver dyads.

Age was hypothesised as a potentially important factor influencing the description of uncertainty during interviews. For example, younger participants might have more concerns about care of children and the effect on work and career. Older participants could have other priorities including maintaining independence and the ability to care for grandchildren or ailing spouses or partners. The disruption caused by a cancer diagnosis could be moderated by the stage of life at which it occurs.

## Methods

### Ethics statement

The study received ethics approval from London Queen Square ethics committee (ref: 15/LO/1323; 14th September 2015). Signed informed consent was obtained from all participants.

### Eligibility and recruitment

Patients with advanced (defined as stage III or IV) ovarian or lung cancer or advanced malignant melanoma and their nominated primary informal caregiver (person who is their main source of support) were identified and approached by the clinical teams at four sites. The cancer types were selected to represent a range of potential experiences of living with advanced cancer. For the same reasons, no restrictions were placed on time since diagnosis or past or current treatment.

Eligibility criteria were 18 years old or over, able to read and speak English and give fully informed consent. Patients were ineligible if they could not nominate an informal caregiver who was also willing to take part. We applied stratified purposive sampling [30] by cancer type (ovarian, lung, melanoma) and age cohort (≤50, 51–65 and ≥66 years) (see S1).

### Procedure

Patient-caregiver dyads were interviewed in their own homes, separately, by two interviewers. Semi-structured interviews lasted approximately 45 min–1 h, were conversational in tone with the pace and duration guided by the participant and were audio-recorded and transcribed verbatim.^1^ Interview topic guides (see S2) were informed by our earlier systematic reviews [31, 32] and through discussion with advisors with a lived experience of cancer or who were supporting someone with cancer. Topics about the impact of extended cancer survival on broader aspects of life and wellbeing included, but were not limited to: how the family was functioning and changes in role responsibilities within the family; impact on occupation and career aspirations and progression, including role responsibilities; finances including loss of income and out of pocket expenses; and leisure and social activities.

Transcripts were reviewed and used to inform subsequent interviews. Reflexive notes were kept to record systematically contextual details of the interviews.

### Analysis

We applied the framework approach to thematic analysis to promote systematic, rigorous and transparent analysis [33, 34]. Two of the authors (VS/RS) read the transcripts and developed the thematic framework from an initial process of open coding. The framework was applied to the transcripts, indexed by themes and subthemes using NVivo 11™ software to facilitate data management. Twenty five percent of material was indexed by two authors (VS/RS) to check for consistency. Themes and subthemes with greater than 2% disagreement between reviewers and where kappa was <0.4 were considered to have unacceptable reliability, suggesting a difference in interpretation between researchers. Content of these themes was reviewed by both researchers and differences in interpretation were resolved through discussion. Redundant subthemes were merged into other themes. The framework was tested iteratively as new transcripts were indexed. The data were then extracted and summarised in charts grouped by themes and subthemes, incorporating field and reflexive notes where appropriate. The charts were used to compare and contrast within and between individual interviews, dyads and patient and caregiver groups. Researchers met regularly to discuss and challenge analytical interpretations.

### Findings

#### Participants

Forty patient-caregiver dyads were invited to participate in the study. Interviews were conducted with 24 patients and 23 caregivers (1 caregiver was too distressed to participate on the day of interview). Reasons for non-participation included: caregiver unwilling/unable to participate (4 dyads), death or poor health of patient (3 dyads), ‘too much going on’ (2 dyads), ‘not interested in study’ (1 dyad) and reasons unknown (5 dyads). If patients did not volunteer a reason for saying no, we did not push for an explanation. One further patient believed she was not eligible for the study having read the participant information sheet. Participant characteristics are presented in Table 1. All patients had stage III or IV disease. In total, 38.5 h of recorded interview was transcribed. Thematic analysis to fulfil the primary objective of item development identified 20 major themes and 33 subthemes. One recurrent topic was ‘uncertainty’ encompassing subthemes such as planning for the future, providing for one’s family, employment and finances (Table 2). The topic of uncertainty and the areas of its influence are explored in greater depth in the analysis described below.

**Table 1:** Participant characteristics

	Overall (*N* = 24)		Ovarian (*N* = 9)		Melanoma (*N* = 9)		Lung (*N* = 6)	
	Patient	Caregiver	Patient	Caregiver	Patient	Caregiver^a^	Patient	Caregiver
Age range (median)	39–84 (62 years)	19–85 (54 years)	44–84 (64 years)	32–85 (54 years)	37–69 (59 years)	36–70 (53 years)	53–79 (63.5 years)	19–68 (54 years)
Male/female	8 (33%) / 16 (67%)	8 (35%) / 15(65%)	–	3 M 6F	6 M 3F	3 M 5F	2 M 4F	2 M 4F
Time since diagnosis	<1 year = 11 1–2 years = 5 >2 years = 8		<1 year = 5 1–2 years = 1 >2 years = 3		<1 year = 3 1–2 years = 2 >2 years = 4		<1 year = 3 1–2 years = 2 >2 years = 1	
Relationship	Spouse/partner = 14 Mother–adult Child = 5^b^ Siblings = 2 Friends = 2		Spouse/partner = 3 Mother–adult Child = 3 Siblings = 2 Friends = 1		Spouse/partner = 8		Spouse/partner = 3 Mother–adult Child = 2 Friends = 1	

^a^Melanoma caregiver *N* = 8 as the wife of one patient was too distressed on the day of interview to continue

^b^All five mother-adult child relationships were adult children acting as caregivers to their mothers

**Table 2:** Themes relating to uncertainty

Topic area	Theme heading	Description	Examples^a^
Jobs and finances	Concerns around employment	This theme incorporates both psychological and physical uncertainties participants express about the patient’s ability to return to work and the relative importance of that	*I’ve got to take the bull by the horns and go back to work and see what I can do and what I can’t do* P71 F44 yrs. *It’s the worst thing possible for somebody like me to be sitting back doing nothing. I just can’t do it. I just can’t put up...I can’t do it. That’s why I’m wanting to get better so I can get myself working again.* P30 M79 yrs
	Loss of earnings	Naturally linked to concerns around employment, participants express specific concerns about supporting their family and their own financial future if they or their loved one are unable to work	*We’ve spent some of our savings, I’ve had to dip into them, and I can’t replenish them because I don’t earn enough money now.* P44 M54 yrs. *I used to manage because there was always overtime there, and we managed as a family. But now I just think, oh my God.* P57 F53 yrs
	Perceived financial position	Financial security is an important moderator of concerns for the future. Participants who feel secure are mindful of the stress for others less fortunate	*We are extremely lucky, we do know that because at least we can still earn an income and we don’t have the financial worries that some people have to have.* P43 F61 yrs. *Being the only one responsible for a house, finances are so important. Um, and I know, as far as I can tell at the minute, I shall be on my own once mum and dad are gone, so to be able to be financially secure is very, very important.* C37 F58 yrs
Relationships and communication	Patient-caregiver relationship and communication	Here, participants discuss supporting each other and the different approaches they employ to maintain ordinary, routine relationships. Sometimes this means trying to protect the other individual by not openly discussing concerns	*I couldn’t manage without him. There’s two of us, we’re a team.* P20 F69 yrs. *We never really talked too much about it. He would say you must tell me everything that you’re feeling so I can help, but I didn’t because I knew it would worry him too much, and he’d be panicking* P12 F64 yrs. *I’m not exaggerating but it is overwhelming. It’s obviously more overwhelming for her than it is for anybody else but it is overwhelming because it’s all consuming and everything is, as far as she’s concerned, it’s all narrowed in on cancer and looking at whether it’s diet, exercise, you name it.* C12 M66 yrs
	Prevalence of cancer conversation	Within this sub-theme, participants describe being exhausted by the ‘cancer conversation’ but not being able to talk about anything else	*I don’t think there’s probably a time when we don’t mention it* P35 F55 yrs. *Yes, I think probably from the fact that you don’t want to spend the whole time talking about it, and by that I really say [my wife] doesn’t want to. And I tend to now think of sentences that I will use that are implying ‘thank you for enquiring but don’t worry’, if you know what I mean, so that they’ve been nice to ask, they are concerned, but let’s not dwell on it.* C20 M70 yrs. *As much as I love them all, it is a bit tiring, every time something new happens I think, oh I’ve got to make phone calls, I’ve got to text, and you’re repeating yourself all the time. It’s a very selfish thing, but it’s tiring, so tiring, to a point where I forget who I’ve told.* P18 M51 yrs
	Family dynamics	Here, participants talk about family function, support, the need to protect loved ones and concerns for their future	*I say to people, cancer doesn’t define you as a person, I wouldn’t let it ever take me down, there’s not a chance, I’m still me and I will always be me, but it definitely changes the whole relationship, the house, the family and the dynamics of everything.* P28 F50 yrs. *Well your children obviously matter, matter probably more than anything in the world, that they’re happy […] that I’m prepared, they’re prepared that I might go early because I know especially two out of the three it’s going to really impact them hard.* P35 F55 yrs
Implications for the future	Changes in outlook, realigning priorities	This is a very positive subtheme, characterised by participants taking stock of their lives and making adjustments, realising what is actually important to them and taking steps to maximise time spent with family, for example, conscious of the precious nature of time	*But I think at the end of it I’ll look back on it and I’ll be like thank god for it. I’ll be like thank god that happened because it has made me stronger and it has made me more knowledgeable and more understanding* C57 F19 yrs. *You realise what’s important actually and that recruitment job’s not really important actually in the scheme of things you know so in the scheme of things the most important things are probably you know you realise your family, friends, spending time and and stuff like that so. Yeah, my outlook on life’s different. You know, and what I want out of life actually* C35 F32 yrs
	Life on hold	Participants feel in limbo, unable to move forward and uncertain how to proceed. For caregivers, this can be linked to feelings of guilt for thinking about their own future	*I think career aspect-wise I am putting that on hold. There’s no way I can get a … would want to really start a career that I really do enjoy with this over my head.* C62 M23 yrs. *I suppose the mistake that I think I have made is I kept trying… It’s like I kept waiting for a difficult period to end so I could reinsert my previous bodily life back in, rather than actually just trying to adapt to this mayhem that I find myself in. I think that’s probably the mistake I’ve made.* C5 F41 yrs
	Opportunities lost	Participants describe the things they feel cancer has stopped them from doing or achieving and the subsequent sense of loss they experience	*I’ve had no retirement, all the things I would like to do I can’t do. I mean, I would have loved a little dog, I can’t take a dog on, not in this situation, it wouldn’t be fair. But places I’d like to go to visit, all of that I feel has gone, and that’s quite hard to deal with. My life’s become quite small, I suppose.* P9 F64 yrs. *I don’t think I really wanted kids, but if I had have done, between mum being sick and [my sister] I’ve gone from 36 to 42. You know, all these things just sort of whizz past and you lose.* C5 F41 yrs
	Not planning for the future	Here participants talk about the inability to plan for both small and large events resulting in a life lived day by day	*Every day I wake up and think “Jesus I’ve got cancer, there’s no cure for it” but as much as maybe I’m even more accepting of that nowadays and I realise that I must enjoy the present and I do do that. But I’m aware of what’s around the corner potentially* P17 M59 yrs. *Because it is just like a big cloud hanging over the top* P71 F44 yrs
	Mortality and death	Uncertainty impacts all aspects of life but underpinning this is the patient’s health and how long it can be maintained	*He took a caveman attitude, she’s going to die, she’s going to leave me, what am I going to do? I’m going to put up this barrier so nobody is going to get to me. But he gradually learnt okay, I’ve got this illness but it doesn’t mean to say I’m going tomorrow.* P43 F61 yrs. *There is no point waiting and thinking we will do that in two years’ time, we will do that in five years’ time because actually no we need to do it now* C44 F53 yrs
Managing uncertainty	Control	Participants link the uncertainty of the situation with feelings of a lack of control. Linked to preserving normality, they describe the importance of not allowing cancer to control their lives	*While ever I can keep strong I am in control of it […] I think it’s very important because I don’t want to feel that it’s getting the better of me, I want to be in control of it, which of course you can’t be, but I do try* P9 F64 yrs. *I am trying to be controlled because if I allowed myself to open up to whatever pathway is going to be his destination then I think I would be overwhelmed […] I think if you let yourself look, and then you think the grandkids won’t see their granddad, and you can’t, that’s not healthy, so I don’t look* C44 F53 yrs
	Preservation of or return to normality	Trying to maintain ‘normal’ activities as a mechanism to cope with the unpredictable situation participants find themselves in	*My priority, in a nutshell, is living life day to day as it’s always been. I don’t make too much room for this, I don’t want to make room for it, I just want to carry on living our lives as we’ve always lived them until such time as we’re presented with a set of situations that precludes that. That’s my priority, is to keep living our lives as they’ve always been.* C46 F65 yrs. *I think you’ve got to, within reason, you’ve got to carry on with your own life otherwise I think that leads to trouble frankly. As things progress, the disease is not going to go away, clearly as things progress that will change but as we stand at the moment, that’s what I believe.* C12 M66 yrs
	Hope	Hope is more passive than the previous two themes. Participants note the insecurity of their position but rather than take active steps to manage it they place their faith in external forces, such as science, doctors, luck and occasionally God	*If you can also meet people, like I often have in the chemo unit, they will ask me what I’ve been through, ‘Oh how can you have done that?’ I say well you’ve just got to say the longer you live more might come up in experiments, and that you can have.* P12 F64 yrs. *Hopefully we caught it early and that’s why, because when I had this mole cut out and it went to here that’s when I first went to see [the consultant], and she said you will be with me for a long time, and I am now, ages.* P44 M54 yrs
	Mindset	Linked to the three previous themes, participants describe facets of their disposition which determine how they approach their tenuous situation	*When you get told you’ve got cancer you know you’re going to die, so just it all depends what you want to do with your life. You can sit and wallow in it, or you can get on with your life and just live your life basically.* P15 M68 yrs. *We were determined that we were not going to be beaten by this, well not easily, I know it will catch us in the end but we’ll go down fighting.* C28 M51 yrs

^a^N.B. these quotes have been selected as generic examples of themes which feed into ‘uncertainty’, not as specific examples of uncertainty per se

Where verbatim quotes are included, codes with a prefix P are quotes from patients, those with a C are caregivers. Participant sex (M/F) and age are also indicated. Square brackets containing three dots […] indicate short sections of omitted speech. Square brackets containing text indicate that a person or place name has been replaced.

We present the findings for patients and caregivers together as the more salient aspects of uncertainty affect both participant groups, and many of the impacts described were broadly aligned. Where meaningful differences between the groups were identified, we have discussed in the text.

#### The pervasive nature of uncertainty

Patients and caregivers alike described feeling a lack of control because they did not know, and no one could tell them, what was going to happen.

It’s something you can’t control. I mean why I find it hard, most things in your life even if you hit a bad patch […] you can control to an extent […] This, you can do nothing; it’s totally out of your control P35 F55yrs

This uncertainty resulted in their lives being lived on a day by day basis, which was deeply unsettling; there was no conceivable way to make plans or look too far in advance. Feeling unable to plan beyond the present was widespread, in stark contrast to discussion about planning for the long term future.

Yes, today not tomorrow. I could never look forward, not not look forward but I don’t think about what’s going to happen tomorrow. I always think about what’s going to happen today. I wake up, how is she? C56 M66yrs

But I suppose I deal with it my way because I just do today, I don’t think two months down the line, or six months, I just deal with it on a daily thing so I don’t get too overwhelmed in my brain trying to process things C44 F53yrs

This resulted in some relatively small life adjustments, such as not booking holidays too far in advance and much bigger life changes in terms of work, finance, family and retirement.

As I say, I just live day-by-day but we don’t plan on booking a holiday in advance […] because it’s not good booking a year in advance P44 M54yrs

Concerns around recurrence, for example, led to an inability to move forwards with life and this uncertainty was observed as a constantly changing landscape, where one aspect was resolved only to be replaced by another.

And then it slowly dawns over time that it’s never going to go away. They mythical all clear is actually never really there because you’re always looking over your shoulder again, constantly aware of what could be there. P26 M37yrs

The inability to plan for the future could became tantamount to feeling there was nothing to look forward to anymore.

Across our interviews, participants described trying to regain control over the uncertain situation. By far the most common strategy was to try to lead as ‘normal’ a life as possible by attempting to maintain home, work and leisure routines, only engaging with cancer when appointments or treatment demanded.

I think it's very important to just go right, okay, this is the way life runs and we just keep doing what we do. Until there's a reason why we can't do what we do, otherwise we’ll just do what we do C18 F53yrs

This allowed them to feel that their lives were not entirely controlled by the cancer. Normality was used not only as an attempt to regain control but also to protect an individual or individuals from what was happening.

Like I said, cancer can define you if you let it but you can make the conscious decision to actually say, do you know what, thumb your nose at it and say no, I’m not going to take any notice P28 F50yrs

#### Uncertainty in relation to work and finances

The significance of work for patients was twofold. First, paid employment obviously affected the ability to provide for themselves and their families. Taking practical steps to ensure that loved ones would have financial stability now and in the future was a driving force.

They said don’t worry, your job’s safe. But you do worry don’t you? P57 F53yrs

Second, was the uncertainty and anxiety around returning to work due to concerns that they might not have the physical ability to do so and changes to the job role if one did together with what might happen if there was a recurrence later. Despite a need to return to work to pay the bills, priorities were sometimes re-evaluated in the light of cancer leading to conflict as to whether work in general or their job in particular was a desirable way to spend time.

Do you want to do that now [work]? Is that fulfilling in your life? P5 F47yrs

Caregivers had a different perspective on work and finances, linked to future consequences of current actions. For example, a willingness to reduce or stop work in order to provide more support if needed, but an awareness of the implications this might have for their own lives further down the line. The potential increasing care needs of the patient posed a serious concern.

Personally I need to keep working to keep my house and if I do have to give up work that’s when the struggle for me internally is going to really happen. Because then I shall want to give up work but I know I will lose an awful lot. That’s going to be hard to face. C37 F58yrs

And it will be quite hard if it’s going to take a lot of time to help look after her. It is going to be really hard because I’ve got so many other things going on in my life. C27 F46yrs

Preserving career goals was important to caregivers to prevent cancer overwhelming their lives entirely and could also be an important part of their identity, separate from the disease.

#### Family life

The future wellbeing of family was a more prevalent concern for patients than for caregivers. For patients with young families, their concern around the future of their family was inextricably linked with jobs and finances and the uncertainties previously described around those. Providing for one’s family and ensuring their material security was a fundamental part of what defined them as a parent. For those patients whose children had grown up, the focus was on how the family would manage emotionally and trying to protect them from distress.

I just want them to be happy and safe and stable and at ease with everything. That’s a big concern for me. P18 M51yrs

I think that for me the security of the family is really important. That’s my role, that’s my job. To think that I might not be there or I might be unable to work and that sort of stuff was really important. P26 M37yrs

Two patients whose caregivers were their adult children expressed concern that their child’s life was essentially on hold. They worried about the future of their children, emotional and financial, and regretted the impact that their illness was having on their lives.

I feel like sometimes they’re putting their lives on hold waiting for me to die, but they don’t quite know when that’s going to be and since I don’t know when it’s going to be and nobody else seems to know when it’s going to be, it’s a bit awkward. P62 F59yrs

#### Uncertainty and retirement plans

Changes to retirement plans and, in particular, giving up plans to move abroad were upsetting. For those who felt this most acutely, it was much more than the home; it was about the dreams they had for retirement and the work they put in to get there, only to have it taken away from them.

Because it wasn’t a holiday destination, it’s our home. It’s our home […] I miss it so much, I really do. It was such a dream. It was such a dream that we had and we achieved it. P17 M59yrs

Spousal caregivers expressed a sense of loss for the future they had anticipated spending with the patient. This could be associated with feelings of selfishness, as if they should not be concerned about apparently trivial things such as travel in the circumstances. There was no blame associated with this loss, just disappointment.

So in retirement one would hope, all other things be equal, that you can travel a bit more, you can see places […] we haven't done that and we're unlikely to do it to be frank, at all […] it's particularly disappointing for me frankly because there are things that I wanted to do, places I wanted to see. C12 M66yrs

#### Uncertainty and non-specific hopes and dreams

The uncertainty of their situation could rob participants of a sense of agency. Rather than focusing on specific things that they were now unable to do or plan for, the cancer had taken their choices away from them.

So yes, you feel a bit cheated I suppose, you can’t do the things you really would have liked P9 F64yrs

For example, participants described possibly wanting to move house, wanting to buy a holiday home, the possibility of moving closer to a grandchild, of travelling more.

We had so many plans […] we talked about buying a place [abroad] for instance but that’s just not going to happen C21 M67yrs

One caregiver described how she had not had a relationship in years due to her caregiving role and that she was now unlikely to have children. She did not know if she wanted to have children, but she felt that choice had been taken away by the cancer because it had eaten away the years.

Unlike the participants who had definite plans for their retirement that had been dashed, these aspirations were not fully formed. What these participants seemed to be mourning was the choice to have done these things. This was sometimes contrasted, perhaps unrealistically, with ‘non-cancer’ families whom they perceived to have the freedom and time to make choices and plans for the future.

Cancer is about what it takes away from you P5 F47yrs

#### Uncertainty drives a sense of ‘life on hold’

Caregivers particularly experienced a sense that their own life was suspended in some way. Like patients, they experienced a lack of control, an inability to know what the future holds for them or make any plans for themselves; however, unlike the patients, they were aware that, at some future point, their lives would resume without the patient in it.

As such, caregivers expressed concern that supporting their loved one made their own financial and working future more precarious. They were mindful of the need to protect their own future and conscious that eventually, they would have to deal not only with the loss of their loved one but also pick up the pieces of their own financial, work and personal life. For this group of caregivers, it was not only what they are giving up now, but also what impact this might have for them in the future.

But I think it’s just that uncertainty, do I stay here for like the next six years or five years or seven years or whatever or even a year and a half or something like that, or do I go and live my own life? Or are we just stuck in the same thing? C62 M23yrs

I do feel like life is on hold to be honest, yes. I think until we get a good period of time of clear results, no operations and things, I think maybe then we might start to think about moving or a new car but I do feel like we’re just stagnant at the moment. C26 F36yrs

Internal conflict arose around the uncertainty surrounding duration of survival and whether it might be easier to have a definite timeframe for death. These thoughts were accompanied by guilt, for even considering their own future and/or future securities without the patient; however, a definite timeframe was also viewed as an opportunity to make the most of time together.

It’s like if you think the person’s going to die, like with mum, it was easier because you were in the today world and it was the end. With [my sister] the whole year we’re moving between, do we have tomorrow, do we not? And that’s really very, very, difficult C5 F41yrs

We have nice times together, we have memories, you have time with daddy, you do this, you do that, and it would be a plan and that would be good in some ways because then we would have a start, beginning, middle and end. At this rate we don't know. So yes, it would be easier because you could then, even if it wasn't a definite, you would roughly have a rough idea and you knew what you were doing C18 F53yrs

#### Talking (or not) about uncertainty—contrasts within dyads

Talking openly about concerns for the future was rare within dyads. More prevalent were descriptions of deliberately *not* talking about it as a means of self-preservation or the preservation of the other person.

There were quite a few tears at first, on my own, don’t tell [my wife] that because I’m supposed to be a stubborn brave sod. P44 M54yrs

By putting it to the back of their minds apart from hospital appointment times, dyads could compartmentalise the cancer to stop it intruding into their everyday. This in itself was a way of maintaining normality and exerting some control over the impact of the disease.

While some dyads colluded in this approach:

A problem shared is a problem doubled for me because then I have to worry about them worrying and I’d rather not. P26 M37yrs

Yes, so that was that conversation literally in the car on the way home from the appointment and then since then we haven't talked about it again. So I know he's thinking about it but, no, generally we don't talk about it C26 F36yrs

For others, it could cause difficulty if one party would like to talk more but the other is closed off:

I know he’s so worried about the cancer, I don’t want him to sound off and say ‘Oh no, leave that, don’t worry about that’ and I’ll say ‘no, I need to talk about it, we need to talk’ P12 F64yrs

Put it in a box and keep the lid on and only let it out when it really needs to be let out you know? C12 M66yrs

Dyads described different views on how best to cope with the uncertainty of their situation. For example, one caregiver wanted to use their savings to enjoy their time together, while the patient wanted to pay off the mortgage to ensure the future security of his family.

For me, we must make the most of every day and not be sitting here waiting for another dollop of bad news to fall in our laps, just go out and enjoy ourselves and do things, go places. We don't want any leftover regrets do we? I have a bucket list, he doesn't, you see, so we're quite different C46 F65yrs

[He] is so cautious and so he’s the one that’s trying to pull me back. I’d go on holidays because you just don’t know what’s going to happen but [he] just thinks about the future and just wants everything to be stable C26 F37yrs

These different approaches to managing the situation could lead to frustration. She (the caregiver) wished he would take more control of the situation, almost as if confronting the issues would in some way affect the outcome:

I want you to do that because I want you to take hold of it. You need to take hold of it and be more in control of it, as much as you can be in control of something that’s horrendous C18 F53yrs

Whereas he (the patient) described a process of acceptance that had incrementally crept up on him as one thing has moved on to another:

I probably have accepted it but there wasn’t a moment of clarity, it was just probably a general slow process of, because it was in stages; everything that’s happened so far has been in stages P18 M51yrs

#### The moderating roles of age and degrees of separation on the impact of uncertainty

Age seemed to moderate some of the uncertainty experienced; cancer was perhaps not encountered as a contingent life event in the same way by older and younger participants. On a practical level, older participants were already retired, and as such, practical concerns around employment and finances were not generally affected as there had been no change since diagnosis.

Alongside the practical aspects, for many of the older participants, there seemed to be a more philosophical acceptance of their situation and indication that they felt they had lived full lives rather than seeing cancer as an unexpected foreshortening of life. There was an absence of discussion of unfulfilled life goals that had been specifically interrupted by the cancer.

I look at life from the point of view that we’re all gonna die of something anyhow […] don’t let it get on top of me at all. P52 F74yrs

The extent to which caregivers experienced their lives to be ‘on hold’ was related to the degree of separation between them and the patient. Those participants who had close familial relationships to the patient and, crucially, lived with them (spouses/partners, some adult children and siblings) experienced the cancer to be much more disruptive to their current and future life than those who were family members living elsewhere or friends.

I can go home and through the front door and, different problems, but here they’re with <her> 24/7. C71 F46yrs

## Discussion

Coming to terms with advanced cancer poses a counter-intuitive problem for a number of participants who originally took their diagnosis to mean that they would die quite quickly, but instead have to adapt to a much less defined future. This adjustment process affects caregivers in equal but subtly different ways. For those who felt that they ‘knew what they were facing’, it was seemingly easier to tackle the future in a more practical and tangible way. For others, there was too much insecurity to be able to formulate an approach to the way forward.

Our findings are in line with other qualitative studies where patients and caregivers have discussed the importance of keeping life normal, the difficulty with long-term planning and the unremitting slog from appointment to appointment, scan to scan with the associated anxiety and uncertainty about the future. [23, 35].

Across our interviews, uncertainty for the future was a predominant theme encompassing issues such as providing for one’s family, employment and finances and plans for retirement. Both patients and caregivers felt a lack of control and an inability to make plans, resulting in a sense that their lives could only be lived on a day-by-day basis removing their sense of agency. There were differences between the groups in the way that the uncertainty manifested discomfort. Patients were particularly concerned with their family’s future financial and emotional wellbeing. Caregivers often felt that their lives were ‘on hold’. Some felt it would be easier to have a definite time frame about death and were frustrated that no one could predict what would happen and when. Similar issues for caregivers, including planning for the future without loved ones and feeling guilty for doing so, feeling stuck in the present and unable to move forward have been reported elsewhere [26].

In our study, age appeared to moderate some uncertainty. In younger participants, there were practical concerns around employment, finances and family. For much older participants, practical concerns around employment and finances were naturally less subject to change, but there was also an absence of discussion of unfulfilled life goals interrupted by the cancer. Reverting to the earlier discussion of cancer as a contingent life event [28, 29] that is, something that happens to an individual which conflicts with their goals and expectations in life, it is a logical supposition that those who consider that they have already lived a full life and are content with their situation, would find cancer less disruptive to their world view and expectations than those who have unfulfilled goals.

Participants around retirement age, however, keenly felt the loss of the future they had worked hard to spend together. We had anticipated that the uncertainty surrounding advanced cancer might have varied effects on patient-caregiver dyads at different stages in life, but were surprised that some of the greatest descriptions of loss were around plans couples had made for their retirement. There is a difference between uncertainty about the ability to do things one *wants* to do such as plans for retirement, travel, moving abroad and those things one *has* to do such as return to work and provide for your family. The former is more of a sense of loss, while the latter perhaps a source of anxiety or distress. Our interviews suggest that we should not underestimate the significance of retirement plans and the real sense of loss experienced if the uncertainty about the future course of disease means that these are put aside.

The sense of ‘loss of future plans’ described by our patients and caregivers sits well with the concept of anticipatory grief i.e. the emotions that relate to loss arising before the event. Originally proposed during the Second World War to describe the depression and loss experienced by wives whose husbands were away fighting [36], the concept is now much broader and while still encompassing the anticipation of a loved one’s death can also be thought to incorporate the anticipating loss of things one wanted for one’s life [26].

The patients and caregivers in our study rarely discussed their concerns for the future with each other and more commonly described *not* talking openly as a means of preservation of self or the other person. This is in line with previous research suggesting that sometimes dyads avoid such conversations to prevent distress [37, 38]. Coyne and Smith [39] describe the coping strategy ‘protective buffering’ by which a person tries to protect their partner from upset by hiding their own worries and concerns, with potentially negative psychological impact [40, 41]. One outcome of not talking about things is that dyads may have differing views on the best way to tackle uncertainty, or lack understanding of the impact for the other person. Our interviews suggest that just because two people are living with the same situation does not necessarily mean that they view it from the same perspective and avoiding conversation, however well-intentioned the reasons, can exacerbate this. Caution is warranted however as we did not set out to measure protective buffering or any other kind of coping strategy; we can only infer from what was discussed in our interviews.

In the introduction, we stated that uncertainty was not a new topic in cancer but that the rapidly changing treatment context for patients makes it timely to revisit the issue here. We reiterate that the added value of this study comes from several aspects of the design which allow a broader understanding of how uncertainty invades many aspects of the patient and caregiver’s life and responsibilities and is not solely concerned with disease status and prognosis. Because we did not specifically prompt participants to talk about feelings of uncertainty, the illustrations are more personal and diverse. For example, we had not anticipated the importance placed on plans for retirement such as moving abroad, but the uncertainty around this future was deeply upsetting for participants.

### Limitations

Like all qualitative studies with small sample size, we must acknowledge that the participant characteristics may limit how broadly our findings can be applied. For example, two thirds of participants in each group were female. Similarly, 14/23 caregivers were spouses/partners. While this likely reflects the balance of caregiving relationships in the wider population, the experiences of people in different relationships was reflected less, and as noted, the impact on the current and future life of caregivers was mediated by the closeness of the relationship and cohabitation. We also chose not to control for treatment type (present or past) or time since diagnosis, although the range of health status and treatments received in the patients we interviewed suggests good representation of different situations. Finally, we should acknowledge that these patients and caregivers are facing extremely challenging circumstances and those who declined to be interviewed may well have been in a poorer state psychologically. We found that of those dyads who declined study, it was often the caregiver that declined rather than the patient and in the interviews conducted, it was the caregivers’ accounts that were more emotional.

## Implications for practice and future research

We had good representation of current health status and past and present treatment in our sample. However, to examine the impact of uncertainty across the disease trajectory in more detail, future research might consider using a longitudinal design.

Social factors such as support from family and friends are likely to contribute to patients’ and caregivers’ ability to cope with the uncertainty of their future and a better understanding of the role of the broader social network may help us to understand why some people cope better than others [42–44]. Similarly, personal factors such as resilience and a higher sense of coherence may be associated with coping with and/or accepting feelings of uncertainty associated with extended survival and may potentially be amenable to intervention [35]. Future research should explore if and how these factors are related to the levels of uncertainty experienced by patients and caregivers and how they manage that uncertainty.

It is usually extremely difficult for clinicians to provide accurate prognostic information to this group of patients which might help them to manage their concerns about the future [45]. Previous research suggests that use of Patient Reported Outcome Measures or even a small number of ‘trigger questions’ in the clinical setting can serve as a ‘conversation opener’ [46, 47], providing patients and their families with an opportunity to discuss concerns in more detail. This has potential to provide an opening for early if not preventive intervention rather than waiting for other psychological sequelae to become established. A similar approach has been applied to facilitate discussion about disease progression and end of life using the Patient Dignity Inventory [48] as the focus of a clinical interview [49]. Further research is required to investigate whether the measures developed in PROACT, or trigger questions derived from them, could function in such a way to highlight some of the issues around uncertainty.

Numerous studies have reported interventions to help cancer patients and their informal caregivers which focus on communication (e.g. [49, 50]), psychoeducational programmes (e.g. [51, 52]) and distress reduction (e.g. [53, 54]). It is possible that early screening and intervention to help dyads discuss their concerns around the future and uncertainty may have the potential to benefit both parties and their relationship together. We already know for example that poor Health Related Quality of Life in one spouse, particularly depressed mood, can adversely affect the partner, both from patient to caregiver and vice versa (e.g. [55, 56]), and screening for distress has been shown to benefit communication and enhance referrals to appropriate support services [57]. Our interviews suggested that members of a dyad sometimes approached the uncertainty of their situation differently. Helping dyads to see the other person’s perspective can facilitate communication which could mediate other positive effects [23]. Further research is therefore required to determine whether a specific intervention to target communication around uncertainty is warranted or whether components of existing interventions could be adapted [58, 59]. Similarly, further investigation should consider whether patients and caregivers experience uncertainty differently and at different times in the trajectory of disease and whether this affects their relationship (e.g. [60]).

## Conclusions

Patients and their informal caregivers face many challenges not only in coping with the prospect of death but also dealing with the uncertainty about survival and the lack of a defined outcome. This uncertainty impacts many areas of life including employment, retirement and general planning for the future. The range and scale of the ‘impact of uncertainty’ varied; however, few people were unaffected by the discomfort of ‘not knowing’. Dyads seldom discussed these concerns with each other, so it might benefit from professional help aimed at facilitating open discussion together.

## Electronic supplementary material

Supplementary File S1 (DOCX 11 kb)

Supplementary file S2 (DOCX 13 kb)

## Footnotes

1 One participant did not wish to be recorded; detailed notes were made during the interview instead and these were coded in the same way as the verbatim transcripts.

## References

[1] Haylock PJ (2010). Advanced cancer: emergence of a new survivor population. Semin Oncol Nurs.

[2] Amir Z, Neary D, Luker K (2008). Cancer survivors' views of work 3 years post diagnosis: a UK perspective. Eur J Oncol Nurs.

[3] Rasmussen DM, Elverdam B (2008). The meaning of work and working life after cancer: an interview study. Psychooncology.

[4] Bradley S (2007). I could lose everything: understanding the cost of a brain tumor. J Neuro-Oncol.

[5] Amir Z (2012). The meaning of cancer: implications for family finances and consequent impact on lifestyle, activities, roles and relationships. Psycho-Oncology.

[6] Timmons A, Gooberman-Hill R, Sharp L (2013). “It’s at a time in your life when you are most vulnerable”: a qualitative exploration of the financial impact of a cancer diagnosis and implications for financial protection in health. PLoS One.

[7] Bailey EH (2010). Impact of multiple caregiving roles on elevated depressed mood in early-stage breast cancer patients and same-age controls. Breast Cancer Res Treat.

[8] Buchbinder M, Longhofer J, McCue K (2009). Family routines and rituals when a parent has cancer. Fam Syst Health.

[9] Hirschman KB, Bourjolly JN (2005). How do tangible supports impact the breast cancer experience?. Soc Work Health Care.

[10] Nijboer C (1999). Determinants of caregiving experiences and mental health of partners of cancer patients. Cancer.

[11] Girgis A, Lambert SD (2009). Caregivers of cancer survivors: the state of the field. Cancer Forum.

[12] Girgis A (2013). Physical, psychosocial, relationship, and economic burden of caring for people with cancer: a review. J Oncol Pract.

[13] Glajchen M (2004). The emerging role and needs of family caregivers in cancer care. J Support Oncol.

[14] Northouse LL (2010). Interventions with family caregivers of cancer patients: meta-analysis of randomized trials. CA Cancer J Clin.

[15] Kim Y (2006). Psychological adjustment of cancer caregivers with multiple roles. Psychooncology.

[16] Fletcher BS (2012). The cancer family caregiving experience: an updated and expanded conceptual model. Eur J Oncol Nurs.

[17] Weitzner MA, Haley WE, Chen H (2000). The family caregiver of the older cancer patient. Hematol Oncol Clin North Am.

[18] Cameron JI (2002). Lifestyle interference and emotional distress in family caregivers of advanced cancer patients. Cancer.

[19] Thomas C, Morris SM, Harman JC (2002). Companions through cancer: the care given by informal carers in cancer contexts. Soc Sci Med.

[20] Kim Y, Given BA (2008). Quality of life of family caregivers of cancer survivors: across the trajectory of the illness. Cancer.

[21] Stamataki Z (2015). Assessing the impact of diagnosis and the related supportive care needs in patients with cutaneous melanoma. Support Care Cancer.

[22] Molassiotis A (2011). Living with multiple myeloma: experiences of patients and their informal caregivers. Support Care Cancer.

[23] Mosher CE (2016). Family caregiving challenges in advanced colorectal cancer: patient and caregiver perspectives. Support Care Cancer.

[24] Mosher CE (2013). Distressed family caregivers of lung cancer patients: an examination of psychosocial and practical challenges. Support Care Cancer.

[25] Hendriksen E (2015). Worried together: a qualitative study of shared anxiety in patients with metastatic non-small cell lung cancer and their family caregivers. Support Care Cancer.

[26] Olson RE (2014). Indefinite loss: the experiences of carers of a spouse with cancer. Eur J Cancer Care (Engl).

[27] Miller LE (2012). Sources of uncertainty in cancer survivorship. J Cancer Surviv.

[28] Kruizinga R (2013). The life in sight application study (LISA): design of a randomized controlled trial to assess the role of an assisted structured reflection on life events and ultimate life goals to improve quality of life of cancer patients. BMC Cancer.

[29] Kruizinga R, et al. Modes of relating to contingency: an exploration of experiences in advanced cancer patients. Palliat Support Care. 2016:1–10. 10.1017/S1478951516000936 27995821

[30] Patton MQ (2002). Qualitative research and evaluation methods.

[31] Shilling V, et al. Patient-reported outcome measures for cancer caregivers: a systematic review. Qual Life Res. 2016. 10.1007/s11136-016-1239-0 PMC4945691 26872911

[32] Catt S, et al. Patient-reported outcome measures of the impact of cancer on patients' everyday lives: a systematic review. J Cancer Surviv. 2016. 10.1007/s11764-016-0580-1 PMC5357497 27834041

[33] Ritchie J, Spencer L, Bryman A, Burgess R (1994). Qualitative data analysis for applied policy research. Analyzing qualitative data.

[34] Pope C, Ziebland S, Mays N (2000). Qualitative research in health care. Analysing qualitative data. BMJ.

[35] Engeli L (2016). Resilience in patients and spouses faced with malignant melanoma. A qualitative longitudinal study. Eur J Cancer Care (Engl).

[36] Lindemann E (1944). Symptomatology and management of acute grief. Am J Psychiatr.

[37] Manne SL (2005). Partner unsupportive responses, avoidant coping, and distress among women with early stage breast cancer: patient and partner perspectives. Health Psychol.

[38] Cordova MJ (2001). Social constraints, cognitive processing, and adjustment to breast cancer. J Consult Clin Psychol.

[39] Coyne JC, Smith DA (1991). Couples coping with a myocardial infarction: a contextual perspective on wives' distress. J Pers Soc Psychol.

[40] Manne SL (2007). Protective buffering and psychological distress among couples coping with breast cancer: the moderating role of relationship satisfaction. J Fam Psychol.

[41] Langer SL, Brown JD, Syrjala KL (2009). Intrapersonal and interpersonal consequences of protective buffering among cancer patients and caregivers. Cancer.

[42] Litzelman K, Kent EE, Rowland JH (2016). Social factors in informal cancer caregivers: the interrelationships among social stressors, relationship quality, and family functioning in the CanCORS data set. Cancer.

[43] Ellis KR (2017). The influence of dyadic symptom distress on threat appraisals and self-efficacy in advanced cancer and caregiving. Support Care Cancer.

[44] Lien CY (2009). Perceived uncertainty, social support and psychological adjustment in older patients with cancer being treated with surgery. J Clin Nurs.

[45] Fallowfield LJ (2017). Therapeutic aims of drugs offering only progression-free survival are misunderstood by patients, and oncologists may be overly optimistic about likely benefits. Support Care Cancer.

[46] Greenhalgh J (2013). How do doctors refer to patient-reported outcome measures (PROMS) in oncology consultations?. Qual Life Res.

[47] Groff S, et al. Examining the sustainability of screening for distress, the sixth vital sign, in two outpatient oncology clinics: a mixed-methods study. Psychooncology. 2017. 10.1002/pon.4388 28128894

[48] Chochinov HM (2008). The patient dignity inventory: a novel way of measuring dignity-related distress in palliative care. J Pain Symptom Manag.

[49] Mowll J (2015). A preliminary study to develop an intervention to facilitate communication between couples in advanced cancer. Palliat Support Care.

[50] Bowman KF (2009). Family caregiver engagement in a coping and communication support intervention tailored to advanced cancer patients and families. Cancer Nurs.

[51] Titler MG, et al. Effectiveness of implementing a dyadic psychoeducational intervention for cancer patients and family caregivers. Support Care Cancer. 2017. 10.1007/s00520-017-3758-9 PMC5610667 28612157

[52] Dockham B (2016). Implementation of a psychoeducational program for cancer survivors and family caregivers at a cancer support community affiliate: a pilot effectiveness study. Cancer Nurs.

[53] Lo C (2015). Managing cancer and living meaningfully: study protocol for a randomized controlled trial. Trials.

[54] Germino BB (2013). Outcomes of an uncertainty management intervention in younger African American and Caucasian breast cancer survivors. Oncol Nurs Forum.

[55] Litzelman K, Green PA, Yabroff KR (2016). Cancer and quality of life in spousal dyads: spillover in couples with and without cancer-related health problems. Support Care Cancer.

[56] Kershaw T (2015). The interdependence of advanced cancer patients' and their family caregivers' mental health, physical health, and self-efficacy over time. Ann Behav Med.

[57] Mitchell AJ (2013). Screening for cancer-related distress: when is implementation successful and when is it unsuccessful?. Acta Oncol.

[58] Northouse LL (2007). Randomized clinical trial of a family intervention for prostate cancer patients and their spouses. Cancer.

[59] McCaughan E (2013). A randomized controlled trial of a self-management psychosocial intervention for men with prostate cancer and their partners: a study protocol. J Adv Nurs.

[60] Borneman T (2014). Death awareness, feelings of uncertainty, and hope in advanced lung cancer patients: can they coexist?. Int J Palliat Nurs.

