# Supplementary material for: The pervasive nature of uncertainty—a qualitative study of patients with advanced cancer and their informal caregivers
Source: J Cancer Surviv. 2017 Jul 18;11(5):590–603. doi: 10.1007/s11764-017-0628-x (PMC5602354; doi:10.1007/s11764-017-0628-x)
Supplement: Supplementary file 1 — (DOCX 11 kb) [file 11764_2017_628_MOESM1_ESM.docx]

**Supplementary File S1: stratification by age group and tumour type**

|  | Lung | Ovarian | Melanoma | Total |
| --- | --- | --- | --- | --- |
| ≤50yrs | 0 | 3 | 2 | 5 |
| 51-65 | 3 | 3 | 4 | 10 |
| ≥66 | 3 | 3 | 2 | 8 |
| Total | 6 | 9 | 8 | 24 |
