# Supplementary material for: The pervasive nature of uncertainty—a qualitative study of patients with advanced cancer and their informal caregivers
Source: J Cancer Surviv. 2017 Jul 18;11(5):590–603. doi: 10.1007/s11764-017-0628-x (PMC5602354; doi:10.1007/s11764-017-0628-x)
Supplement: Supplementary file 2 — (DOCX 13 kb) [file 11764_2017_628_MOESM2_ESM.docx]

**Supplementary file S2: summary topic guides**

All participants, patient or caregiver, were asked to discuss issues around:

- **Family life**

Including: communication; changes in role responsibilities within the family such as caring responsibilities; role reversal/shift {e.g. parent looked after by child}

- **Relationships**

As for family life with specific probe for relationship with nominated caregiver

- **Jobs and careers**

Including: (If still working) changes in roles and/or responsibilities; plans to return to work; impact on career progression and/or aspirations

- **Financial wellbeing**

Including: loss of income (if appropriate); benefits; out of pocket costs; long term planning/savings

- **Leisure and social activities**

Role of hobbies and social life in quality of life

- **Psychological wellbeing**

Including: confidence; self-esteem; outlook on life

- **Physical functioning (e.g. ability to perform usual tasks)**

Including: ability to perform usual activities; the role of physical health in quality of life; and impact on independence

These areas were discussed in terms of: importance to participant; change, if any, since diagnosis; any adaptations they had made to maintain (if important); and any knock on to other related aspects of life.

Participants were also asked to generate their own topics important to their wellbeing and discuss in the same way.
